# Physicians’ Visiting List for 1854

**Published:** 1853-12

**Authors:** 


*Physicians' Visiting List for* 1854.—Lindsay & Blakiston have published this annual convenience, and Derby, Orton & Mulligan have it for sale in this city. Its clean pages are suggestive of long lists of paying patients, whose names may some day be inscribed thereon. What a record of suffering would the history of those names afford! S. B. H.

